# How I Speak Defines What I Do: Effects of the Functional Language Proficiency of Host Country Employees on Their Unethical Pro-organizational Behavior

**DOI:** 10.3389/fpsyg.2022.852450

**Published:** 2022-03-17

**Authors:** Ya Xi Shen, Chuang Zhang, Lamei Zuo, Xingxing Zhou, Xuhui Deng, Long Zhang

**Affiliations:** ^1^Business School, Hunan University, Changsha, China; ^2^Institute of Facility Agriculture, Guangdong Academy of Agricultural Sciences, Guangzhou, China

**Keywords:** language, multinational corporation, organizational identification, moral disengagement, unethical pro-organizational behavior, knowledge transfer

## Abstract

Functional language has been used in many multinational corporations (MNCs) as a way to overcome the problems caused by the coexistence of multiple languages in the workplace. The existing literature has explored the importance, adoption, and effectiveness of functional language. Yet, how functional language shapes host country employees’ moral cognition and behavior is insufficiently researched. Guided by the Social Identity Theory, this manuscript shows that host country employees’ functional language proficiency (i.e., English) enhances their unethical pro-organizational behavior through their linguistic group identification and moral disengagement. We tested our predictions using the data collected from 309 full-time host country employees through an online survey, and the results generally supported our hypotheses. The findings make contributions to both international management and language literature and organizational moral behavior literature.

## Introduction

With the development of international business, an increasing number of multinational corporations (MNCs) have established subunits around the world. When headquarters and subsidiaries are situated in different linguistic zones, multiple languages are often used by MNC employees (Cohen and Kassis-Henderson, 2017; Vigier and Spencer-Oatey, 2017). Language, as the basis of communication, is essential to the understanding of organizational processes. The use of multiple languages can be the origin of many communication problems that occur at different organizational levels. For instance, the use of multiple languages leads to tension between headquarters and subsidiaries (Vaara et al., 2005; Fredriksson et al., 2006). Moreover, the lack of a common language may result in a misunderstanding between top management and general employees (Barner-Rasmussen and Aarnio, 2011), as well as in the segregation of employees who speak different first languages. Therefore, most MNCs introduce a single functional language policy, aiming to overcome the possible problems associated with interorganizational languages and the facilitation of communication within organizations.

According to Luo and Shenkar (2006), functional language can be defined as “the language formally designated for verbal and written use by an MNC’s focal unit (headquarters or overseas subunit) within this unit and with the rest of the MNC network.” As Bialystok (1981) suggested, functional language focuses on the use of language for conversations, information, etc. Additionally, Dong et al. (2018) highlighted the contextual and practical use of functional language. Several studies (e.g., Piekkari et al., 2005) have illustrated the importance of functional language for MNCs and their employees. From an organizational perspective, Harzing and Pudelko (2014), for instance, claimed that English as a business lingua franca helps shorten the language distance within an MNC. Functional language may also contribute to trust building (Feely and Harzing, 2003) and tacit knowledge inflow (Reiche et al., 2015) between headquarters and subsidiaries. From an MNC employee perspective, the use of functional language has also been illustrated as affecting their workplace experiences, such as their social status (Hinds et al., 2014), leadership positions (Paunova, 2017), and interpersonal relationships (Henderson, 2005) in the organizational context. Therefore, whether employees can speak the functional language and how proficiently they do so may directly or indirectly impact their work experience.

**FIGURE 1:**
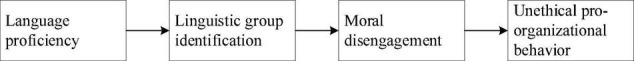
Proposed mediated model.

Employees may be segregated into different groups based on their various functional language proficiencies. Some levels of communication may involve only employees who speak the functional language at a proficient level, thereby excluding the remaining employees who lack this proficiency (Fantini, 1995; Selmer, 2006). The inclusion and exclusion of employees based on their language proficiency may further impact their attitudes toward the organization as well as their organizational behaviors. For instance, these employees may be more likely to proactively participate in organization-related activities and protect their organizational interests despite moral criteria because they have positive emotions toward the organization owing to their language-based “in-group” status (Sharoni et al., 2015). In this case, research has shown that employees’ “in-group” status within the organization often motivates them to perform activities that disregard their ethical standards to defend their organization’s interests, leading to workplace misconduct and unethical behavior (Babatunde and Viet, 2021). Based on the relationship between language proficiency and linguistic identity and the moral disengagement that may arise from employee identification and the subsequent unethical behavior it triggers, the model proposed in this manuscript is shown in Figure 1.

The purpose of this study is to explore how functional language proficiency impacts host country MNC employees’ moral disengagement and unethical pro-organizational behavior (UPB) in MNC host country subunits. This research field is important but has been overlooked for at least four reasons. First, the process of language use has been neglected in the areas of international business and MNC management for many years (Marschan et al., 1997). Most MNCs ignore the complexity of language and simply introduce a single functional language policy as the unified answer for all organizational language problems (Feely and Harzing, 2003). However, whether an organization should use a single functional language may be only the starting point. How functional language is used in organizations may be more important, as it focuses more on the actual language use process and the problems that may occur during this process. However, most existing studies focus on the “whether or not” part of functional language, for instance, the willingness to adopt a functional language (Bordia and Bordia, 2015), the effectiveness of using a single functional language (Fredriksson et al., 2006), and the strategy of building a better functional language policy (Luo and Shenkar, 2006). Very few studies have focused on exploring the actual process of using a functional language. Given the reality that single functional language policies are already widely used in most MNCs around the world and that this current status cannot be changed easily, at least not in a short time. Currently, it is more meaningful to look at “how well” functional languages have been used (e.g., proficiency). It is also important to understand how the functional language use process may impact employees’ work, non-work experience, and organizational behavior and attitudes instead of focusing only on “whether or not” the functional language should be used.

In addition, the existing body of literature examining functional language use has typically focused on organizational headquarters. From a global perspective, the majority of management researchers are from Western (e.g., English) backgrounds in which most MNCs are founded; therefore, these researchers are less likely to notice and understand the problems that host country MNC employees may face. Their language studies are more likely to focus on the controlling function when taking an organizational approach (Feely and Harzing, 2003; Reiche et al., 2015) and on expatriates’ language use experience when taking an employee approach (Zhang and Harzing, 2016). Although several studies have explored the problems encountered by host country MNC employees when using functional languages at work, host country MNC employees as a group are under-researched in the current literature on language and international business. However, this group plays an important role in MNCs. Host country MNC employees account for a large portion of all MNC employees, and their performance has a great influence on overall MNC performance as well as that in the local area. The organizational behavior and attitudes of these employees toward MNCs are therefore worth researching.

Third, few studies have explored functional language proficiency from an identity perspective. In the handful of studies that have focused on functional language proficiency, functional language speaking has been studied mostly as a communication skill (Selmer, 2006) and, in some cases, as a specific status characteristic based on status characteristics theory (Paunova, 2017). However, functional language can also be understood from an identity perspective. Linguistic identity is an integral part of individuals’ social identity (Bordia and Bordia, 2015; He et al., 2020; Wu and Chen, 2021). Individuals define themselves and others partly through their language use. In MNC subunits, whether host country MNC employees have a functional language linguistic identity and how strong this identity is guide their attitudes and behavior toward their colleagues as well as toward the organization that defines the functional language. Therefore, functional language proficiency should also be studied through a social identity pathway.

Finally, moral disengagement has emerged as a key mediatory mechanism, but little is known about how it links linguistic identification with organizational behavior and attitudes toward MNCs. Research has shown that when employees increase their identity with the organization, they engage in more ethical defensive behaviors toward the organization, including the use of unethical tactics and wrongdoing. In addition, moral disengagement has been shown to be positively associated with workplace misconduct and negatively related to organizational citizenship behaviors (Babatunde and Viet, 2021). However, the role played by moral disengagement in the relationship between linguistic identity and employee behavior has not been adequately studied.

Having identified these key research gaps and illustrated their importance to both the broader language literature and organizations, this article aims to explore how functional language has been used by host country MNC employees (whose first language is not the functional language) and how this experience may impact their work outcomes in MNC subunits (where the local language is different from that of the headquarters). A conceptual framework is then proposed to explain how functional language proficiency impacts linguistic identity and how linguistic identity impacts unethical behavior through moral disengagement. Social Identity Theory is used as the overarching theory to inform the model development.

## Theoretical Background

### Social Identity Theory

Social identity theory is used as the overarching theoretical perspective to guide the model development. On the one hand, Tajfel (1972) defined social identity as “*the individual’s knowledge that he belongs to certain social groups together with some emotional and value significance to him of this group membership*” (p. 292). On the other hand, social identity generally refers to individuals’ feeling of whom they are based on the social group or category to which they belong (Hogg and Abrams, 1988). In other words, the core idea is that individuals distinguish themselves and others based on the various social groups to which they belong. To maintain their self-esteem, individuals tend to focus on the positive aspects of their own groups and negative aspects of other social groups and then reject out-group members (Tajfel and Turner, 1986). Additionally, existing theory shows that in-group members may lower their ethical standards to help protect their positive views of the group, ultimately increasing unethical actions within the group (Kundro and Nurmohamed, 2020).

According to Tajfel et al. (1979), one’s social identity is formed through a three-stage process—social categorization, social identification, and social comparison. The social categorization stage is mainly the “what people have” stage, in which individuals create social groups based on what people have (e.g., gender, skills, or nationality) and categorize people into different groups, the core idea of which is that these different attributions dominate in various groups (Li et al., 2021). The identification stage then focuses on “who people are” or “who people want to be.” In this stage, individuals attach themselves to specific groups. They adopt the cultures and norms of those groups and express themselves as group members. Social identification can help people decrease uncertainty in organizational settings through the “identity prototype” (Porck et al., 2019). Then, the last stage is the “how people behave” stage. In the social comparison stage, people behave based on their identification with certain social groups; they compare their own groups with others to distinguish themselves from other people, and they exaggerate and accentuate their in-group similarities and out-group differences, carrying out positive activities for their own group, and negative activities for other groups to maintain their self-esteem. Moreover, social comparison may affect team performance by influencing people’s behavior (Lam et al., 2011).

Linguistic identity refers to individuals’ attachment to certain linguistic groups. As people always define themselves and others based on their language use (Giles, 1977), linguistic identity is an integral part of their social identity (Bordia and Bordia, 2015). Therefore, based on the three-stage process of social identity theory, what language individuals speak and how well they master it (social categorization) will impact their identification with a certain linguistic group (social identification). As the functional language plays an important role in the working communication of many MNCs (Dong et al., 2018), its linguistic identity can be considered part of MNCs’ organizational identity. The use of an organizational functional language within MNCs may lead to the creation of a new social group within the MNC subunits in host countries. Only employees who speak the functional language proficiently belong to this group (Seyranian, 2014), while local employees who cannot speak the functional language or speak it awkwardly may be excluded (Hogg and Abrams, 1988). Therefore, according to the three-stage process of Tajfel et al. (1979), identification with a functional language group should enhance one’s organizational identification (social identification), which then impacts an individual’s attitude and behavior toward the organization (social comparison) (Karelaia et al., 2021). For example, high social comparison requires employees to take the organization’s perspective; they regard organizational goals or values as their own. Therefore, when faced with a moral dilemma, these employees would rather engage in unethical behavior than say anything negative about the organization or undermine its interests (Valle et al., 2019).

### Language Proficiency and Linguistic Group Identification

In MNC subunits where the functional language is different from the local language, some MNC employees speak the functional language more proficiently than they do other languages (Janssens et al., 2004; Peltokorpi and Vaara, 2012). Previous studies have explored functional language proficiency mainly from a communication skill perspective by arguing that the more proficient an employee can speak the functional language, the better communication skill he or she has in the working context, and therefore, the easier he or she is able to obtain the necessary information in the organization, build better relationships with colleagues, and fit into the organizational culture (Takeuchi et al., 2002). As previously mentioned, the effect of functional language on employees’ organizational behavior may also be explored from the identity perspective, yet few studies have focused on it. In this conceptual manuscript, we consider functional language proficiency from an identity perspective. According to social identity theory, individuals attach themselves to different social groups based on their social identity (Tajfel, 1972). Therefore, we propose that individuals will identify with the functional language linguistic group if they speak the functional language (Kroon et al., 2015; Reiche et al., 2015).

Functional language linguistic identity differs from inborn identities (e.g., nationality, first language, or gender) in which the adoption and development of identities occur naturally. Whether to adopt foreign functional language and how proficiently one speaks the second language is a choice of the individual itself. This decision may affect the attitude of individuals toward identity. If individuals prefer a functional language that is different from their inborn language, then they may have negative attitudes toward their inborn identities and therefore weakly identify with those social groups (Bordia and Bordia, 2015), they will always hold positive attitudes toward the identities with which they choose to associate, or otherwise lack the motivation to develop those identities in the first place. Therefore, people are more likely to identify with the social groups that contain their acquired social identities (Zhang et al., 2017; Karhunen et al., 2018; Zhang et al., 2021), as they believe that belonging to these social groups can improve their self-esteem. In addition, the more effort that individuals devote to developing these acquired social identities, the stronger their social identities are, and the more likely they are to identify themselves as being members of these social groups. Therefore, we hypothesize:

***Hypothesis 1:*** Host country MNC employees’ functional language proficiency is positively related to their functional language linguistic group identification.

### Functional Language Linguistic Group Identification and Moral Disengagement

For MNC subunits where functional language is used, functional language can be considered part of organizational identity (Ashforth et al., 2008). Ashforth et al. (2008) portrayed some content of the identity that plays an essential role in the organizational identification process, developing a broad three-layer formulation of identification that not only considers the “core of the identity” (e.g., I am and I feel) but also focuses on the “content of the identity” such as the values (I care), the goals (I want), as well as the skills, knowledge, and abilities (I can do) attached to the membership, and finally the “behaviors of the identity” (I behave). Therefore, for host country MNC employees, the ability to speak the functional language is, first, a skill (I can speak the organizational language) that is included in the MNC organizational identity. This ability may also be considered as a value (I care about the organizational culture) because the functional language usually represents the culture of the headquarters at which the MNC was founded. Therefore, the ability to speak the functional language helps explain what it means to be an MNC member. The identification of the functional language linguistic group contributes to the identification of the organization that uses the functional language.

Moral disengagement was first introduced by Bandura (1986), who defined it as “*a set of cognitive tactics that allow people to sidestep moral self-regulatory processes that normally prevent misconduct.*” Many antecedents, such as conscientiousness, trait empathy, and moral identity, lead to individuals’ moral disengagement (Babatunde and Viet, 2021). In the context of the rapid development of MNCs, previous studies have shown that employees’ performance is affected by moral disengagement (Probst et al., 2020; Ogunfowora et al., 2021), but few studies have paid attention to the relationship between linguistic group identification and moral disengagement. Therefore, this study aims to explore this relationship and to take a closer look at the antecedents of moral disengagement. This study explores the positive relationship between individuals’ linguistic group identification and their moral disengagement. As mentioned previously, based on social identity theory, individuals behave positively to benefit their own group during social comparison. Therefore, employees who strongly identify with their organization take its perspective, values, and goals as their own, thus activating their moral disengagement when faced with an ethical dilemma to contribute to the overall benefit of the organization (Van Knippenberg, 2000). In addition, an organization can be considered a social group, and functional language proficiency can be considered a shared or collective identity. The more strongly employees believe in their linguistic-based identification of the functional language linguistic group, the more likely they are to perceive themselves as members of the organizational group, which is referred to as organizational identification, and the more likely they are to enhance their propensity for moral disengagement. Thereby, we propose the following:

***Hypothesis 2:*** Host country MNC employees’ functional language linguistic group identification is positively related to their moral disengagement.

### Moral Disengagement and Unethical Pro-organizational Behavior

According to Babatunde and Viet (2021), high levels of moral disengagement predict a wide range of undesirable outcomes, for instance, non-ethics outcomes, such as turnover intentions, and immoral behavior, such as workplace misconduct. UPB is among the most likely outcomes because it satisfies the demand to maintain self-esteem. As mentioned previously, individuals intend to take actions that jeopardize the out-group but benefit the organization (social comparison). Therefore, we intentionally link moral disengagement with UPB.

How does linguistic identification influence UPB? Based on social cognitive theory, one reason to adopt unethical behavior is that some cognitive mechanisms dampen moral constraints. Individuals all have a self-regulatory mechanism through which moral standards are developed (He et al., 2019). If this mechanism works well, then unethical behavior will be prevented because no individual wants to disobey their moral standards and thus feel guilty. Otherwise, moral constraints are weakened, resulting in unethical behavior (Narwal et al., 2021). Linguistic identification provides the premise on which to simulate moral disengagement. When moral disengagement is activated, host country MNC employees’ self-regulation fails to function well (Baron et al., 2015). By interpreting misconduct, employees’ moral responsibility is weakened, and hence, their feelings of guilt are reduced, which makes them feel okay about doing something morally wrong. Due to this lack of moral constraints, the possibility of these individuals engaging in UPB is greatly increased.

Individuals may engage in unethical acts by employing any one of eight “cognitive maneuvers,” namely, types of moral disengagement (Babatunde and Viet, 2021): moral justification, euphemistic labeling, advantageous comparison, distortion of consequences, displacement of responsibility, diffusion of responsibility, attribution of blame, and dehumanization. In this case, host country MNC employees who have high linguistic group identification and, hence, high organizational identification are more likely to engage in UPB for the sake of MNC interests. These employees may justify this as being acceptable because it serves the organization (moral justification), describe this activity in an innocuous manner (euphemistic labeling), or minimize its outcome by comparing it to organizational loss (advantageous comparison). Therefore, moral disengagement is a key mediatory mechanism through which linguistic identification influences UPB. Thereby, we propose the following:

***Hypothesis 3:*** Host country MNC employees’ moral disengagement mediates the positive relationship between functional language linguistic group identification and UPB.

## Materials and Methods

### Sample and Procedures

We collected our data from Mainland China which has become the second-largest global recipient of foreign direct investment (FDI) and the inflow continues increasing every year (UNCTAD, 2019). Our sample was recruited from full-time host country employees working in Western MNCs’ Chinese subsidiaries. The survey was distributed purely online, and the survey link was sent to participants via a Chinese multifunctional messaging app called WeChat. Surveys were sent to a total of 375 employees, and 309 completed surveys were returned, yielding a response rate of 82.40%.

As suggested by Podsakoff et al. (2012), to reduce common method bias, data were collected at two time points with a 2-week interval. The first survey measured participants’ perceived organizational functional language proficiency (i.e., English proficiency) and their functional language linguistic group identification. The second survey mainly measured participants’ moral cognition and behaviors (i.e., moral disengagement, UPB).

Among the final sample of employees, 43.0% were male [standard deviation (SD) = 0.50], 96.1% of participants had a high school education or above (SD = 0.75), and the average age was 28.60 years (SD = 0.76).

### Measures

#### Organizational Functional Language Proficiency

Self-reports of language proficiency are widely used in language research and are strongly related to objective measures of language proficiency (Marion et al., 2007; Mayberry et al., 2011). Therefore, in this study, we asked participants to rate their language abilities with a single item: “How would you evaluate your English level/fluency?” (*1* = *not good at all, 5* = *perfect*).

#### Linguistic Group Identification

Employees’ linguistic group identification was measured using a four-item version of Luhtanen and Crocker’s (1992) collective self-esteem scale. This scale is divided into four subscales, namely, membership, private, public, and identity subscales. This study chose to use the private subscale and replaced “social groups” with “language groups” to represent the construct of language group identity. An example item is as follows: “I feel good about the language groups I belong to” (*1* = *strongly disagree, 5* = *strongly agree;* α = *0.73*).

#### Moral Disengagement

We measured moral disengagement with three items from Chen et al. (2016). An example item is as follows: “It would be ok to be less than fully truthful to protect [my company’s] interests” (*1* = *strongly disagree, 5* = *strongly agree;* α = *0.89*).

#### Unethical Pro-organizational Behavior

We used six items from Umphress et al. (2010) to measure this construct. An example item is as follows: “If it would help my organization, I would misrepresent the truth to make my organization look good” (*1* = *strongly disagree, 5* = *strongly agree;* α = *0.88*).

## Results

In this study, SPSS 24.0 and Mplus 8.1 were used to preprocess and analyze the data. Regression analysis and stepwise regression, as suggested by Baron and Kenny (1986), were used to verify the hypotheses.

### Preliminary Analyses

Table 1 presents the means and standard deviations of and correlations among the study variables. On this basis, Table 2 shows composite reliability (CR), average variance extracted (AVE), maximum shared squared variance (MSV), and average shared squared variance (ASV) values of the main variables in this study. According to the analysis results, CR values are all greater than 0.7, while AVE values are all greater than MSV and ASV values. Therefore, the constructs in this study exhibit good construct reliability and discriminant validity. Furthermore, to verify the validity of our main theorized model, we conducted a confirmatory factor analysis (CFA) using Mplus 8.1. Table 3 presents the results of our measurement model, which provided an adequate fit to the data (χ^2^ = 279.27, *df* = 62, *p* < 0.01; CFI = 0.90, TLI = 0.87, RMSEA = 0.10, and SRMR = 0.06).

**TABLE 1:** Means, standard deviations, and correlations.

Variable	Mean	SD	1	2	3	4	5	6
1. Gender	1.57	0.50						
2. Age	2.72	0.76	−0.22**					
3. Education	3.08	0.75	0.02	−0.13*				
4. Organizational functional language proficiency	3.30	1.01	0.05	–0.06	0.50**			
5. Linguistic group identification	3.82	0.78	0.11*	–0.01	0.32**	0.64**		
6. Moral disengagement	2.34	0.94	0.10	0.03	0.07	0.18**	0.15**	
7. UPB	2.32	0.81	0.16**	–0.10	0.08	0.09	0.06	0.68**

N = 309, *p < 0.05, **p < 0.01. UPB, unethical pro-organizational behavior.

**TABLE 2:** Results of reliability and validity analyses.

	CR	AVE	MSV	ASV
Linguistic group identification	0.72	0.45	0.41	0.15
Moral disengagement	0.89	0.73	0.03	0.17
UPB	0.88	0.54	0.46	0.16

*CR, composite reliability; AVE, average variance extracted; MSV, maximum shared squared variance; ASV, average shared squared variance; UPB, unethical pro-organizational behavior.*

**TABLE 3:** Results of confirmatory factor analyses.

Model	χ^2^	*df*	CFI	TLI	RMSEA	SRMR
Three-factor model (proposed model)	279.27	62	0.90	0.87	0.10	0.06
Two-factor model: LGID and MD were combined into one factor	623.71	64	0.74	0.68	0.17	0.12
One-factor model: all variables were combined into one factor	789.33	65	0.66	0.59	0.19	0.12

*CFI, comparative fit index; TLI, Tucker-Lewis index; RMSEA, root mean square error of approximation; SRMR, standardized root mean square residual, LGID, linguistic group identification; MD, moral disengagement.*

### Hypothesis Testing

Regarding hypothesis 1, as shown in Table 4, simple regression analysis showed a positive relationship between organizational functional language proficiency and linguistic group identification (Model 1, β = 0.57, *p* < 0.01). Therefore, hypothesis 1 is supported. In addition, we tested our main theoretical model using stepwise regression according to Baron and Kenny (1986). As shown in Table 4, the results illustrate that linguistic group identification is significantly and positively related to moral disengagement (Model 2, β = 0.13, *p* < 0.05). Thus, hypothesis 2 is supported. Hypothesis 3 proposed that moral disengagement plays a mediating role in the relationship between linguistic group identification and UPB. The process results showed that the indirect effect is significant and positive (Model 3, β = 0.69, *p* < 0.01). Therefore, hypothesis 3 is supported.

**TABLE 4:** Results of regression analyses.

Variables	Model 1		Model 2		Model 3		
Gender	0.11	0.09	0.11	0.09	0.14*	0.14	0.08
Age	0.06	0.05	0.06	0.05	–0.06	–0.07	–0.10*
Education	0.33	0.01	0.07	0.03	0.07	0.06	0.04
OFLP		0.64**					
LGID				0.13*		0.03	–0.06
MD							0.69**
*R*	0.34	0.65	0.13	0.18	0.18	0.19	0.71
Adjusted *R*^2^	0.11	0.41	0.01	0.02	0.03	0.02	0.49

N = 309, *p < 0.05, **p < 0.01; In Model 1, Y, Linguistic group identification; In Model 2, Y, Moral disengagement; In Model 3, Y, Unethical pro-organizational behavior; OFLP, Organizational functional language proficiency; LGID, Linguistic group identification; MD, Moral disengagement.

The results of this study clearly show that organizational functional language proficiency has a direct effect on linguistic group identification, which is consistent with the previous research on functional languages (Kroon et al., 2015; Reiche et al., 2015). However, unlike previous studies, this study found a significant positive correlation between linguistic group identification and moral disengagement, thus complementing the literature on these topics. In addition, unlike previous studies that focused mainly on the relationship between linguistic identity and employee performance (Probst et al., 2020; Ogunfowora et al., 2021), this study focuses on the relationship between linguistic group identification and employee UPB. The data analysis results show that moral disengagement plays a mediating role in the relationship between linguistic group identification and UPB.

## Discussion

The purpose of this study is to provide insight into how host country employees’ functional language proficiency shapes their moral cognition and behavior. Guided by the Social Identity Theory and supported by a sample of 309 full-time host country employees, our results revealed the positive effects of host country employees’ functional language proficiency on their linguistic group identification. The results also suggest that moral disengagement acts as an effective mediator between linguistic group identification and UPB. In the following section, we discuss the theoretical contribution, practical implications of this study, as well as the limitations and possible future research directions.

### Theoretical Contributions

First, our framework contributes to the existing language literature by highlighting the importance of functional language use processes in international business and management within the MNC context. This work emphasizes “how” functional language is used rather than “whether” it should be used (Fredriksson et al., 2006). More specifically, our framework introduces language proficiency, that is, how well the functional language has been used by employees. MNCs set subunits in different language zones. To overcome the problems caused by the use of multiple languages, functional language policy is widely used in MNCs (Piekkari et al., 2005). However, the introduction of functional language is not the end of the story. During the day-to-day use process, functional language may have a great impact on employees’ work and non-work experience, attitudes toward the organization, and organizational behavior. We developed the contextual use in definition of functional language proficiency (Dong et al., 2018). This research proposes a model that uses social identity theory to explain how host country MNC employees’ functional language use process impacts their work outcomes both within and outside of MNCs. When employees believe that speaking the functional language can enhance their self-esteem and is important to their roles in their workplace, their functional language group identification contributes to activating moral disengagement and then guides their attitudes and behaviors toward MNCs.

Second, our model also fills the gap in the research regarding the language use experience of host country employees, as this is an important group to be studied. Host country MNC employees account for the majority of all MNC employees, and thus, their performance directly influences the overall performance of MNCs. Functional language proficiency is an issue that mainly influences host country MNC employees (Janssens et al., 2004; Peltokorpi and Vaara, 2012); the functional language chosen is usually the local language of MNC headquarters (e.g., English), thus usually having less impact on headquarters employees or expatriates. Therefore, when setting up a language policy, MNC headquarters are less likely to notice the importance for and impact of the functional language on host country MNC employees (Feely and Harzing, 2003). The model proposed in the manuscript draws attention to language using a process of host country employees, exploring how host country MNC employees’ use of functional language influences their moral disengagement and, hence, their work outcomes.

Third, this article contributes to the existing identity language and international business literature by adopting an identity perspective, rather than communication perspective (Takeuchi et al., 2002; Harzing and Pudelko, 2014). The proposed model focuses on linguistic group identification. First, although there are several other papers focusing on language proficiency (e.g., Selmer, 2006; Paunova, 2017), this study is among the first to study functional language proficiency from a linguistic identity pathway by considering that language proficiency represents the strength of a linguistic identity. Second, through the model, this study illustrates the detailed process of how employees’ linguistic identity leads to their linguistic group identification, moral disengagement, and UPB. Some of the existing related research has illustrated the relationship between language and career growth/improvement (Neeley and Dumas, 2016) but has not explained how this happens and the underlying factors. This study contributes to explaining the link between language use and organizational outcomes. Therefore, it not only represents a new and important angle through which to study the language use process but also expands existing studies by providing the detailed relationship between the language use process and work outcomes.

Fourth, this article contributes to the existing ethics literature by exploring the mediation effect of moral disengagement. We first shed light on the role of context in shaping moral disengagement. Although many aspects of the organizational context can mitigate or exacerbate this key factor (Zhang et al., 2016; Huang et al., 2017; Babatunde and Viet, 2021; Black et al., 2021), this study is the first to explore language identification as an antecedent. We find that moral disengagement is at least partly determined by value or culture in an overall work environment—more specifically, functional language use. Second, we find the key mediatory mechanism effect in an ethical context, which depicts that when facing an ethical dilemma, linguistic identification plays a critical role in influencing employees’ misconduct (e.g., UPB) in a way that impacts how they construe moral choices, thus weakening their self-regulation. Instead of studying the effects on performance (Probst et al., 2020; Ogunfowora et al., 2021), these results support the Bandura et al. ’s (1996, 2001) findings that moral disengagement fosters low prosocialness.

### Practical Implications

The results of this study yield several practical implications for MNCs. Our research suggests that in MNCs, language proficiency has a great influence on employees’ linguistic group identification and ultimately organizational identity, meaning that when employees demonstrate higher language proficiency, they are more likely to see themselves as part of a linguistic group and thus have stronger organizational identity. Functional language proficiency in MNCs equips employees with the ability to communicate and identify groups, which in turn facilitates their effectiveness at work. Since language proficiency is important, at the corporate level, organizations should provide some language training courses to employees. Therefore, managers can enhance the language proficiency of employees by training them in the functional language of the organization, thereby increasing their organizational identity.

Our results also demonstrate that linguistic group identification plays an important role in the effect of language proficiency on host country MNC employees’ UPB. For the sake of the organization, the proper execution of language design decisions is necessary for employees to demonstrate stronger linguistic identification. When employees have a strong sense of identification with the functional language of their organization, they are more likely to adopt pro-organizational unethical behavior to safeguard organizational interests. Thus, on the one hand, managers focus exclusively on enhancing their employees’ linguistic identity; on the other hand, managers must be aware that when the organization uses its functional language to strengthen the identity of employees within the organization, employees should be informed of the need to take a more ethical approach to safeguard organizational interests.

### Limitations and Directions for Future Research

We should note several limitations of the present research, with the potential to generate future research avenues. The first limitation concerns common method bias and causality in the relationships among the variables. There are threats of common method bias (Podsakoff et al., 2012) because all the data of participants in our study are from a single source. On the one hand, although we have been able to demonstrate the mediation model across two-time lagged data, our study, with features of correlational designs, creates difficulties in terms of causal inference, and is inevitably threatened by common method bias. On the other hand, even though the self-reported data approach was deemed appropriate in studying the nature of functional language in the current study, objective language proficiency measures may also be useful in examining the relationship between linguistic identity and employee behavior from different angles. Therefore, we suggest that further experimental or longitudinal studies, with multi-source data obtained using a combination of objective and subjective measures, are needed to test the implied causality with greater confidence.

Second, additional research is needed to investigate contextual boundary conditions. Many personal and situational factors may come into play to determine this relationship. An important boundary condition that warrants future research is the perceived importance of functional language. Although linguistic group identification itself enhances the propensity of employees to take positive actions for an organization and generate unfavorable outcomes regardless of moral standards, people with different extents of perceived importance of functional language may experience different unethical outcomes. Future research may use this characteristic or other boundary conditions to confirm this prediction.

## Data Availability Statement

The raw data supporting the conclusions of this article will be made available by the authors, without undue reservation.

## Ethics Statement

The ethical aspects of this research were approved by the Australian National University Human Research Ethics Committee when one of the authors (YS) was affiliated there before commencement of the study. They were subsequently approved by Hunan University. The participants provided their online informed consent to participate in this study.

## Author Contributions

YS: conceptualization and design of study and methodology. CZ: drafting the manuscript and analysis and interpretation of data. LZu and XD: acquisition of data and revising the manuscript. XZ: investigation and resources. LZh: revising the manuscript critically for important intellectual content. All authors contributed to the article and approved the submitted version.

## Conflict of Interest

The authors declare that the research was conducted in the absence of any commercial or financial relationships that could be construed as a potential conflict of interest.

## Publisher’s Note

All claims expressed in this article are solely those of the authors and do not necessarily represent those of their affiliated organizations, or those of the publisher, the editors and the reviewers. Any product that may be evaluated in this article, or claim that may be made by its manufacturer, is not guaranteed or endorsed by the publisher.
